# Highly Efficient Biotransformation of Phenolic Glycosides Using a Recombinant β*-*Glucosidase From White Rot Fungus *Trametes trogii*

**DOI:** 10.3389/fmicb.2022.762502

**Published:** 2022-05-18

**Authors:** Yuan Qu, Yuan Luo, Xulei Yang, Yu Zhang, En Yang, Huini Xu, Yingying He, Irbis Chagan, JinPing Yan

**Affiliations:** ^1^Laboratory of Bioconversion, Life Science and Technology College, Kunming University of Science and Technology, Kunming, China; ^2^Yunnan Provincial Key Laboratory of Panax notoginseng, Kunming, China

**Keywords:** biotransformation, β-glucosidase, glycosyl hydrolases 3, phenolic glycosides, *Trametes trogii*

## Abstract

Phenolic glycosides are the important bioactive molecules, and their bioavailability can be influenced by enzyme hydrolysis, such as β-glucosidases (EC3.2.1.21) and other glycosyl hydrolases (GHs). Wood rotting fungi possess a superfamily of GHs, but little attention has been paid to the GHs and their potential applications in biotransformation of phenolic glycosides. In this study, two GH3 gene family members of *Trametes trogii* S0301, mainly expressed in the carbon sources conversion stage were cloned, and TtBgl3 coded by *T_trogii*_12914 showed β-glucosidase activity toward 4-nitrophenyl β-D-glucopyranoside (*p*NPG). The recombinant TtBgl3 preferred an intermediately neutral optimum pH with >80% of the maximum activity at pH 5.0–7.0 and was stable at a wide range of pH (5.0–10.0). Phenolic glycosides transformation experiments showed that TtBgl3 was a dual-activity enzyme with both activities of aryl-β-D-glucosidase and β-glucuronidase, and could hydrolyze the β-glucoside/glucuronide bond of phenolic glycosides. Under optimized conditions, the recombinant TtBgl3 had much higher transformation efficiency toward the β-glucoside bond of gastrodin, esculin and daidzin than β-glucuronide bond of baicalin, with the transformation rate of 100 and 50%, respectively. Our homology modeling, molecular docking, and mutational analysis demonstrated that His85 and Lys467 in the acceptor-binding pocket of TtBgl3 were the potential active sites. The point mutation of His85 and Lys467 leads to the significantly impaired catalytic activity toward *p*NPG and also the weak transformation efficiency toward gastrodin. These findings provide insights for the identification of novel GH3 β-glucosidases from *T. trogii* and other wood-rotting fungi. Furthermore, TtBgl3 might be applied as green and efficient biological catalysts in the deglycosylation of diverse phenolics to produce bioactive glycosides for drug discovery in the future.

## Introduction

Phenolic glycosides are formed by the dehydration condensation of aglycon and sugars, widely found in fruits, vegetables, beans, grains, nuts, and other plants (Srinroch et al., 2020; Topaloviæ et al., 2020; Li et al., 2021). Phenolic glycosides mainly include phenol glycosides, coumarin glycosides, and flavonoid glycosides (Rasouli et al., 2017). Studies have shown that phenolic glycosides have multiple pharmacological effects such as anti-oxidation, anti-fatigue, anti-aging, anti-inflammatory, whitening, and anti-cancer (Nugroho et al., 2017; Zhang et al., 2019c). However, the polyhydroxy structure of phenolic glycosides has the disadvantages of low solubility and poor stability in fat-soluble systems (Teng and Chen, 2019). Therefore, polyhydroxy compounds are modified to expand their applications in the food, pharmaceutical, and cosmetic industries. After hydrolysis into aglycones by intestinal flora or enzymes, such as β-glucosidases and β-xylosidase, the hydrophobicity and fat solubility of phenolic glycosides are enhanced. Consequently, they are easier to be absorbed into the blood through the biofilm to exert their efficacy and improve their bioavailability (Ahmed et al., 2017; Teng and Chen, 2019).

For example, isoflavones are structural homologs of human estrogens and exist in legumes as glycosides, especially in soybeans (Franke et al., 1994; Halabalaki et al., 2006). Research shows that isoflavones can prevent some cancers such as breast cancer, prostate cancer, and colon cancer (Ravindranath et al., 2004; Ullah et al., 2016), reduce the risk of cardiovascular disease, improve bone health (Alekel et al., 2015), and so forth. However, the pharmacological effects of isoflavones are not directly related to glycosides but related to isoflavone aglycones such as daidzein and genistein, the absorption of isoflavone aglycones in the human intestine is faster than that of glycosides (Izumi et al., 2000). Similarly, baicalin and baicalein, as the main active substances in *Scutellaria*, have similar pharmacological effects, such as antibacterial, antiviral, and anti-inflammatory (Kubo et al., 1981; Austin et al., 1992; Shen et al., 2003). Baicalein is absorbed faster than baicalin, but its content is lower, making it more difficult to extract. Therefore, enzymatic hydrolysis is a better way to obtain baicalein (Lai et al., 2003a,b). Phenolic glycosides can be absorbed and utilized by the human body through hydrolysis to aglycones (Lai et al., 2003a).

As the important glycosyl hydrolases (GHs), β-glucosidases are mainly present in family GH1 and GH3, and can hydrolyze disaccharides, oligosaccharides, and aryl and alkyl β-glucosides, thus releasing β-D-glucose from the terminal non-reducing ends (Kudou et al., 1991; Florindo et al., 2018; Zhang et al., 2019a,b). Filamentous fungi are the main producers of β-glucosidase. In many fungal strains, β-glucosidase isoenzymes with different expression patterns and physicochemical characteristics have been demonstrated, which makes it possible to explore high-quality β-glucosidase resources. Until now, many β-glucosidase isoenzymes from fungi are successfully expressed in yeasts, *Pichia pastoris* and *Saccharomyces cerevisiae*, or in filamentous fungi (Cummings and Fowler, 1996; Skory et al., 1996; Takashima et al., 1999; Dan et al., 2000). However, the activity of fungal β-glucosidase enzymes depends on the post-translational modifications such as glycosylation, and the high activity expression of fungal β-glucosidase in *E*scherichia *coli* is little (Li et al., 2005). In addition, a majority of recombinant fungal β-glucosidases belong to GH3 in both eukaryotic and prokaryotic expression systems (Ahmed et al., 2017).

*Trametes trogii*, one of the important wood-degrading fungi in nature, can degrade lignocellulose components and also a wide range of toxic environmental pollutants (Izumi et al., 2000; Park et al., 2013; Liu et al., 2019). In a previous study, detailed genomic and transcriptomic analysis showed that *T. trogii* S0301 contained 602 CAZyme-encoding genes in which GHs accounted for 39.53%, which demonstrated the full potential of *T. trogii* as a source of industrial enzymes. However, strains of *Trametes* such as *T. trogii* S0301 are known as the model organisms for AA enzyme production, particularly laccase (Franke et al., 1994; Halabalaki et al., 2006; Liu et al., 2019; Teng and Chen, 2019). Little attention has been paid to the potential application of GHs of *Trametes* strains in bioconversion.

In a previous study, the GH3 gene family was identified in the whole-genome sequence of *T. trogii* S0301 consisting of 10 GH3 gene family members. Based on these results, the main objectives of the present study were to (i) analyze the gene structure and the expression pattern of GH3 gene family members, (ii) purify and characterize the recombinant GH3 TtBgl3 (*T*_*trogii*_12914), and (iii) assess the bioconversion ability of the recombinant TtBgl3 toward different types of phenolic glycosides.

## Materials and Methods

### Fungal Strain

The *T. trogii* S0301 strain was routinely maintained on the GYP medium (2% glucose, 0.5% yeast extract, 0.5% tryptone, and 0.1% MgSO_4_⋅7H_2_O) at 4°C at the Biotechnology Research Center of Life Science and Technology College, Kunming University of Science and Technology (Yan et al., 2014; Zhang et al., 2021).

### Sequence and Transcriptome Data Analysis

The genome and putative mRNA sequencing data of *T. trogii* S0301 strain presented here are associated with NCBI BioProject PRJNA480364 and BioSample SAMN09635320 (Liu et al., 2019). The structure of GH3 gene family members was analyzed using the online software Gene Structure Display Server,^1^ and GH3 protein domains were identified and annotated using the Simple Modular Architecture Research Tool.^2^ BioEdit software was used for sequence alignment, and the software package MEGA 6^3^ was used for phylogenetic analysis. According to the methods described in our previous study, gene expression profiles of the GH3 gene family members were analyzed under the three culturing conditions, Highley’s basal salt medium containing 1% (w/v) glucose (1% G), 0.5% (w/v) glucose, and 0.5% (w/v) ball-milled oak woods (0.5% LG), or 1% (w/v) ball-milled oak woods (1% L), respectively (Liu et al., 2019).

### Gene Cloning and Plasmid Construction

Total RNA was extracted from 6-day-old fresh hyphae of *T. trogii* S0301 on the GYP medium using TRIzol RNA Isolation Reagents (Promega, United States). Then, the first-strand cDNA was synthesized using HiScript II Q RT SuperMix for qPCR (+gDNA wiper) (Vazyme Biotech, China). The open reading frame of TtBgl3 (*T*_*trogii*_12914) was then amplified by PCR from the cDNA with the specific primers 5′-tctagaATGTCGCGCGACTTCCTCG-3′ (TtBgl3-F) and 5′ctcgagCACCCCGTTCCATGTGAATC-3′ (TtBgl3-R). After digesting by *Eco*RI and *Xho*I, the resulting PCR fragments were cloned to the prokaryotic expression vector pET-28b which was previously digested by the same restriction enzymes. After sequencing, the resulting plasmid, named pET-28b-TtBgl3, was transformed into the *E. coli* strain *Rosetta* (DE3) and the single colony containing the cDNA of TtBgl3 was obtained (Zhang et al., 2021).

### Expression and Purification of TtBgl3

The expression of the recombinant enzyme was performed by adding 0.1 mM isopropyl-β-D-thiogalactopyranoside (IPTG) to the bacterial suspension at the initial concentration of OD600 = 0.7. After 18 h at 16°C, the induced cells were harvested by centrifugation at 4°C for 5 min at 10,000 *g* and washed twice with PBS buffer (pH 7.4). After sonication and centrifugation at 12,000 *g* for 10 min, the supernatants was obtained as the crude enzyme solution. To purify the recombinant TtBgl3, the crude enzyme solution was applied onto a Ni-NTA column (Sangon Biotech, China). The TtBgl3 protein was eluted from the column with midazolam buffer by gradient elution. Enzyme fractions were analyzed by sodium dodecyl sulfate polyacrylamide gel electrophoresis (SDS-PAGE) with a 5% (w/v) stacking gel and a 12% (w/v) separating gel. After electrophoresis, SDS-PAGE was stained with Coomassie blue R-250. Protein concentrations were determined using a bicinchoninic acid (BCA) protein assay kit (Tiangen Biotech, China) (Zhang et al., 2021).

### Enzyme Activity Assay

The β-glucosidase activity was determined with 4-nitrophenyl β-D-glucopyranoside (*p*NPG) as the substrate as described previously. In brief, a reaction mixture containing 5 μL of 10 mM *p*NPG and 5 μL of appropriately diluted crude enzyme in phosphate citrate buffer (200 mM, pH 6.0) was evaluated at 50°C for 10 min. And the reactions were stopped by adding 150 μL of 1 M Na_2_CO_3_ solution. Then, the increase in the absorbance at 405 nm was recorded, and one unit of activity was defined as the amount of enzyme that released 1 μmol of *p*NPG per minute under the test conditions. All the experiments were performed in triplicate (Qin et al., 2018; Zhang et al., 2021).

### Effect of pH and Temperature on TtBgl3 Activity

To determine the optimal pH of the recombinant TtBgl3 activity, the reaction mixtures were preincubated with 200 mM disodium hydrogen phosphate-citric acid buffer (pH 2.4–8) at 50°C for 10 min. To determine the optimal temperature of the recombinant TtBgl3 activity, the reaction mixtures were pre-incubated at given temperatures (30–80°C) at the optimum pH for 10 min. For the pH and thermal stability analysis, the enzyme was preincubated with different buffers (pH 3.0–11.0) at 37°C for 2 h or at the optimal pH at 50 and 60°C for different time durations without *p*NPG. Then, the activity of the reaction mixture or the pretreated enzyme was measured under the optimum pH and temperature according to the standard enzyme determination. An identical amount of enzymes placed on ice was used as the positive control and set as 100%. All assays were performed in triplicate (Li et al., 2018).

### Effect of Metal Ions on TtBgl3 Activity

The reaction mixture containing 200 mM disodium hydrogen phosphate-citric acid buffer (pH 5.0), 1 mM *p*NPG and 50 U/mL of TtBgl3, was mixed with Fe^3+^, Sr^2+^, NH^4+^, Ba^2+^, K^+^, Na^+^, Co^2+^, Mg^2+^, Ni^2+^, Ca^2+^, Mn^2+^, Cu^2+^, and Zn^2+^ at the final concentration of 10 mM to determine the effect of metal ions on the activity of TtBgl3 (Akram et al., 2018). After incubation at 50°C for 10 min, the increase in the absorbance at 405 nm was measured according to the standard enzyme determination. The activity of the reaction mixture without metal ions was recorded as 100%.

### Kinetic Study

The kinetic parameters (*K*_*m*_, *V*_*max*_, and *K*_*cat*_) of the recombinant TtBgl3 were estimated in disodium hydrogen phosphate citric acid buffer (pH 5.0, 200 mM) at 55°C for 5 min, with different concentrations of *p*NPG (0.2–1.5 mM) as the substrates. The data were calculated and analyzed according to the Lineweaver–Burk method (Li et al., 2018).

### Biotransformation of Phenolic Glycosides by TtBgl3

We selected phenols (gastrodin), coumarins (esculin), isoflavones (daidzin), and flavonoids (baicalin) as the transformation substrates of TtBgl3 to analyze the transformation ability of the recombinant TtBgl3 to different phenolic glycosides. The reaction mixture (400 μL) consisted of 200 mM sodium phosphate buffer (pH 6.0), 1 mg/mL substrate and 80 U/mL recombinant TtBgl3. After incubating the reaction mixture for 12 h at 37°C, the reaction was stopped using 400 μL of methanol. The mixture was centrifuged at 10,000*g* for 30 min at 4°C. The supernatant was analyzed by UHPLC-ESI-Q-TOF-MS (Agilent Technologies, United States) to confirm the identity of the transformation products. The optimal dosage of the recombinant TtBgl3 was determined by adding 1, 5, 10, 20, 40, 60, and 80 U/mL of the recombinant TtBgl3 to the reaction system for 12 h at 37°C. The optimal transformation time of the recombinant TtBgl3 was determined by incubating the transformation system after adding 10 U/mL recombinant TtBgl3 at 37°C for 0.5, 1, 2, 4, 8, and 12 h. All samples were analyzed by high-performance liquid chromatography (HPLC).

### High-Performance Liquid Chromatography Analysis

All the transformation samples were analyzed by HPLC-UV on a Shimadzu Analytical Instrument (Shimadzu, Japan). The separation was performed on a YMC-pack ODS-A column (4.6 × 250 mm; i.d., 5 μm; YMC Co., Ltd., Japan) using a mobile phase of water with 0.05% phosphoric acid (A) and acetonitrile (B) at a flow rate of 1 mL/min. The isocratic profile of gastrodin was as follows: 0–25 min, 3% B. The gradient profile of esculin was as follows: 0–20 min, 20% B; 20–25 min, 20–50% B. The gradient profile for the separation of daidzin and baicalin was as follows: 0–1 min, 5% B; 1–25 min, 5–70% B. The column temperature was set at 30°C. The detection wavelength of gastrodin, esculin, daidzin, and baicalin was set at 220, 348, 250, and 280 nm, respectively (Yan et al., 2016; Chang et al., 2018).

### Homology Modeling of TtBgl3 Structure and Molecular Docking

Homology modeling of TtBgl3 was performed using SWISS-MODEL software^4^ based on the crystal structure of TtBgl3 (PDB accession number 3abz.1.A). The 2D structures of phenolic glycosides (gastrodin, esculin, daidzin, and baicalin) were obtained from the PubChem database.^5^ The optimized structures of four phenolic glycosides were then used for docking with TtBgl3, using AutoDock Vina (version 1.1.2). The structure was visualized and analyzed using PyMOL.

### Site-Directed Mutagenesis of TtBgl3

The pET-28b-TtBgl3 plasmid was used as the template for site-directed mutagenesis to introduce *H*85*A* and *K*467*L* point mutations into TtBgl3 using a Fast Site-Directed Mutagenesis Kit (Tiangen Biotech, China), with subsequent verification by DNA sequencing. Mutagenic primer pairs were as follows: 5′-CGGAGTTCGCGGCTCCTCTCTCTTCGTTTCCAC-3′ (TU-A-F) and 5′-AGAGAGGAGCCGCGAACTCCGTTTGGGC CGTC-3′ (TU-A-R) for H85A; 5′-GCAAGACACTCGTGTCA TACTGAATGACTTC-3′ (TU-B-F) and 5′-ATGACACGAG TGTCTTGCAGAACGGTCTCC-3′ (TU-B-R) for *K*467*L*. The mutagenic nucleotides are shown in italic type. The expression of the mutant protein and transformation system toward gastrodin was assessed as described previously.

### Data Analysis

The results of all experiments were based on the average of three independent experiments ± standard deviation (SD), and the statistical significance was determined using the unpaired-samples *t*-test and one-way analysis of variance.

## Results

### Glycosyl Hydrolase 3 Gene Family and Their Expression Pattern Under Glucose and Wood Powder as Carbon Source

**FIGURE 1:**
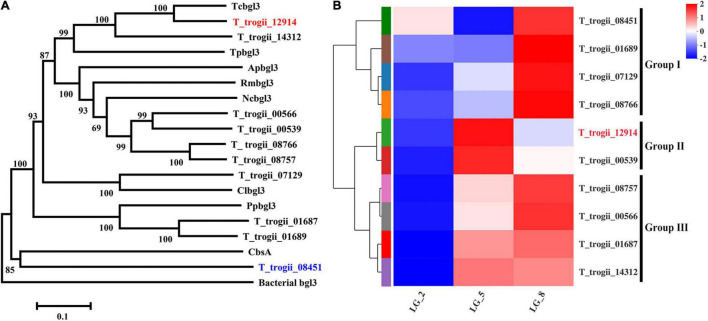
Phylogenetic analysis and the expression profles of the GH3 gene family. **(A)** Phylogenetic analysis of the GH3 gene family members of *T. trogii* S0301. The scale bar represents 0.1 substitutions per site. Phylogenetic analysis was conducted using MEGA6 software. **(B)** The expression profles of GH3 gene family members in GYP medium added with 0.5% lignocellulose and 0.5% glucose.

The *T. trogii* genome contains 10 genes (Supplementary Figure 1) that can be classified as the GH3 gene family (Liu et al., 2019). Multiple sequence alignment (Supplementary Figure 2) and phylogenetic tree analysis showed that the coding sequences of all the identified GH3 genes except *T*_*trogii*_08451 possessed the conserved Glyco_hydro_3, Glyco_hydro_3_C, and Fn3-like domains, and clustered with the putative β-glucosidas, whereas *T_trogii*_08451 lacked the Fn3-like domains and clustered together with CbsA, a member of N-acetyl beta-glucosaminidase (Figure 1A). When cultivated on the GYP liquid medium containing wood powder (0.5%, w/v) and glucose (0.5%, w/v) as the carbon sources (Liu et al., 2019) for 2 (LG_–_2), 5 (LG_–_5) and 8 days (LG_–_8), the expression pattern of 10 predicted β-glucosidases in *T. trogii* S0301 could be divided into three groups. The increase in four GH3 transcripts *T_trogii*_ (08451, 01689, 07129, and 08766) in Group I and four GH3 transcripts (*T_trogii*_08757, 00566, 01687, and 14312) in Group III was in line with the level of glucose consumption and maintained at a higher level during the lignocellulosic utilization stage (LG_–_8), while the transcription of two GH3 transcripts (*T_trogii*_12914 and 00539) in Group II was only up regulated at the point of glucose depletion (LG_–_5) (Figure 1B). These results indicated that GH3 family members of Group II might mainly be involved in the carbon source conversion stage, while members of Group I and III were the enzymes involved in lignocellulose degradation.

### Prokaryotically Expression and Purification of TtBgl3

**FIGURE 2:**
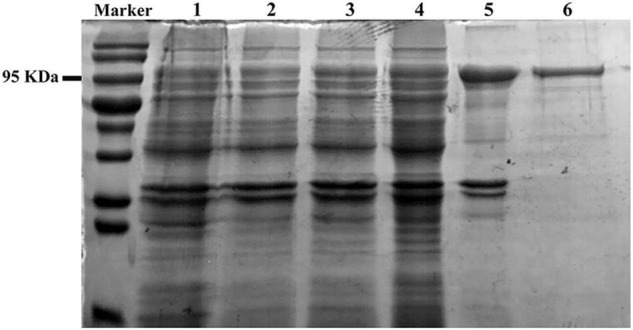
Sodium dodecyl sulfate polyacrylamide gel electrophoresis analysis of the recombinant TtBgl3. The supernatant of the crude extracts of *E. coli Rosetta* (DE3) (Lane 1). The total protein of *E. coli Rosetta* (DE3) harboring pET28b-*TtBgl3* without (Lane 2); the total protein (Lane 3), supernatant (Lane 4), and precipitation (Lane 5) of *E. coli Rosetta* (DE3) harboring pET-*TtBgl3* treated by IPTG at the concentration of 0.1 mM. The recombinant TtBgl3 purified by Ni-NTA resin affinity chromatography and ultrafiltration (Lane 6).

To further explore the characteristics of two GH3 members in Group II, the cDNAs of *T_trogii*_12914 and 00539 were cloned and prokaryotically expressed. The recombinant protein coded by *T_trogii*_12914, with the calculated molecular weight value of 97 kDa (Figure 2), had significant activity toward *p*NPG, and did not exhibit hydrolytic activity toward pNP-GlcA, pNPX, cellobiose, and cellotriose, which further confirmed that *T_trogii*_12914 coded a typical GH3 glucosidase, named TtBgl3. In addition, the expressed proteins coded by *T_trogii*_ 00539 existed in the form of inclusion body and was inactive toward *p*NPG.

### Biochemical Properties of the Recombinant TtBgl3

**FIGURE 3:**
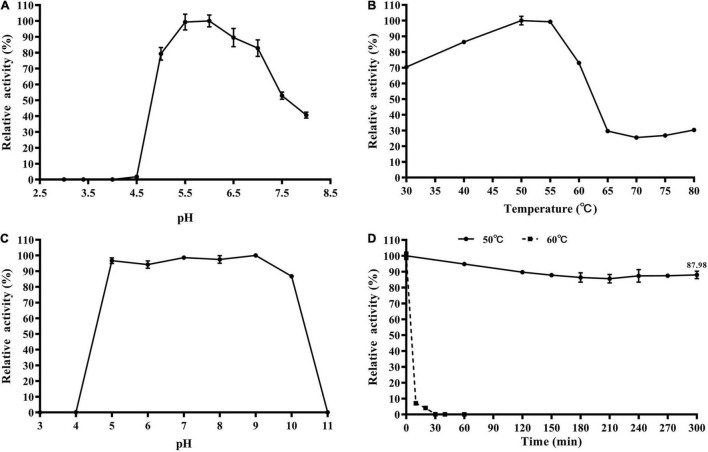
Biochemical characterization of the recombinant TtBgl3. The optimum pH **(A)** and temperature **(B)** of the recombinant TtBgl3 using *p*NPG as substrate. The effects of pH **(C)** and temperature **(D)** on the stability of TtBgl3.

The enzymatic properties of the recombinant TtBgl3 were determined using *p*NPG as the substrate. The optimum pH ranged from pH 5.5 and 6.0, with >80% of the maximum activity at pH 5.0–7.0 (Figure 3A and Table 1). The optimum temperature for TtBgl3 was 50°C (Figure 3B and Table 1). TtBgl3 was stable at a wide range of pH from 5.0 to 10.0 with more than 95% of maximum activity after 2 h of treatment (Figure 3C). TtBgl3 retained 90% of their initial activity after 5 h incubation at 50°C (Figure 3D). However, β-glucosidase activity decreased rapidly at temperatures above 60°C (Figure 3). The specific activity, *K_*m*_, V_*max*_*, and *K*_*cat*_ values of TtBgl3 for *p*NPG at 50°C and pH 5.5 were determined to be 341.5 U/mg, 0.55 mM, 263.16 μM/mg/min, and 164.5, respectively (Table 1). Catalytic efficiency (*K*_*cat*_/*K*_*m*_) of TtBgl3 against *p*NPG were 297.6 (Table 1). TtBgl3 activity increased with Fe^3+^ and decreased with Ni^2+^, Ca^2+^, Mn^2+^, Cu^2+^, Zn^2+^, while ${\text{Sr}}_{3+}^{2+}$, Ba_2+_, K^+^, Na^+^, Ca^2+^, and Mg^2+^ did not show any obvious effect (Table 2).

**TABLE 1:** Properties of the purified β-glucosidase from fungi.

Strain	Enzyme	Specific activity (U/mg)	K_*m*_ (mM)	Vmax (μ M mg^–1^ min^–1^)	*Kca*t	*Kcat*/*K*_*m*_	Optimal condition (Tem/pH)	T_1/2_ (min)	References
*Myceliophthora thermophila*	MtBgl3b	258.7	2.78	927.9	N/A	N/A	60°C/5	>120 min (60°C); >80 min (65°C)	Zhao et al., 2015
*Myceliophthora thermophila*	MtBgl3	97.7	0.39	47.9	N/A	N/A	70°C/5	>240 min (55°C)	Karnaouri et al., 2013
*Fomes fomentarius*	1,4-β-glucosidase	282	62	NA	N/A	N/A	60°C/5	NA	Vìtrovskı et al.,2013
*Talaromyces amestolkiae*	BGL-2	82.6	0.41	NA	485	1167	60°C/4	>72 h (40°C)	Méndez-Líter et al., 2017
*Monascus sanguineus*	β-Glucosidase	7.625	0.89	7.56	N/A	N/A	60°C/5	NA	Dikshit and Tallapragada, 2015
*Gongronella* sp.	BglW5	59.00	0.39	NA	19.6	50.26	70°C/4.5	>60 min (50°C)	Fang et al., 2014
*T. trogii* S0301	Bgl3	341.5	0.55	263.16	164.5	297.6	50°C/6	>120 min (50°C)	This study

**TABLE 2:** Effects of metal ions at the concentration of 10 mM on TtBgl3 activity.

Metal ions	Relative activity (%)	Reagents	Relative activity (%)
Control	100.0	CaCl_2_	94.7
FeCl_3_	119.6	MnSO_4_	90.9
SrCl_2_	102.9	CuSO_4_	85.5
NH_4_Cl	100.4	ZnSO_4_	81.8
BaCl_2_	99.9	NiCl_2_	95.5
KCl	99.2	CoCl_2_	97.5
NaCl	98.2	MgCl_2_	97.1

### Biotransformation of Phenolic Glycosides by TtBgl3

**FIGURE 4:**
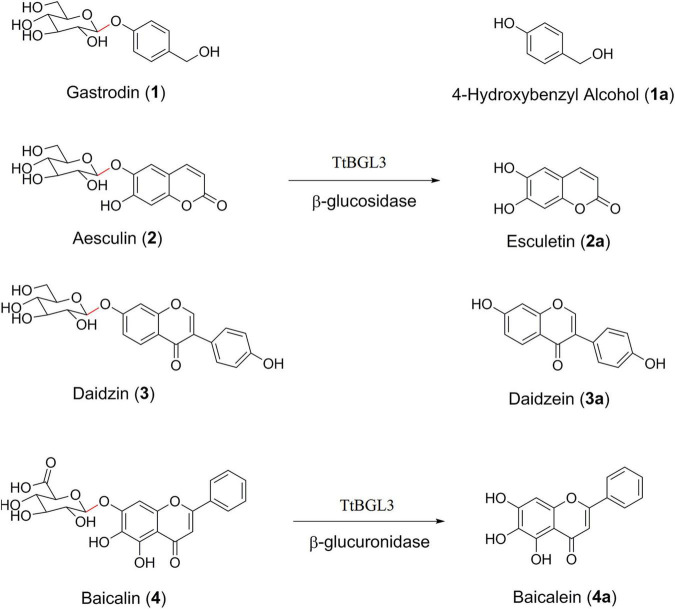
Chemical structures of phenolic glycosides. Gastrodin (1); 4-hydroxybenzyl alcohol (1a); esculin (2); esculetin (2a); daidzin (3); daidzein (3a); baicalin (4); baicalein (4a).

**FIGURE 5:**
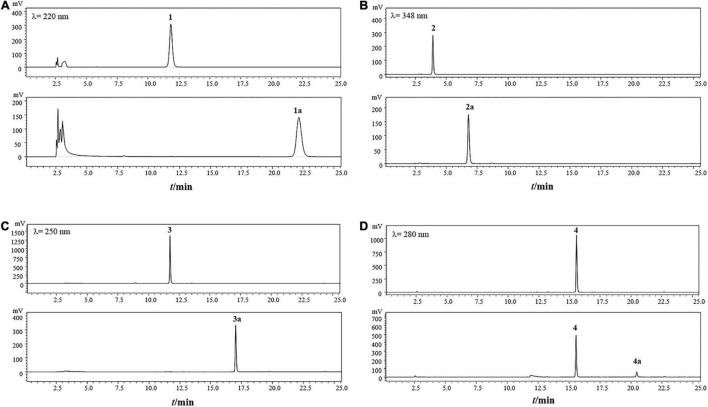
HPLC analysis of the transformation products of gastrodin **(A)**, esculin**(B)**, daidzin **(C)** and baicalin **(D)** by recombinant TtBgl3 for 12 h. Gastrodin (1); 4-hydroxybenzyl alcohol (1a); esculin (2); esculetin (2a); daidzin (3); daidzein (3a); baicalin (4); baicalein (4a).

**FIGURE 6:**
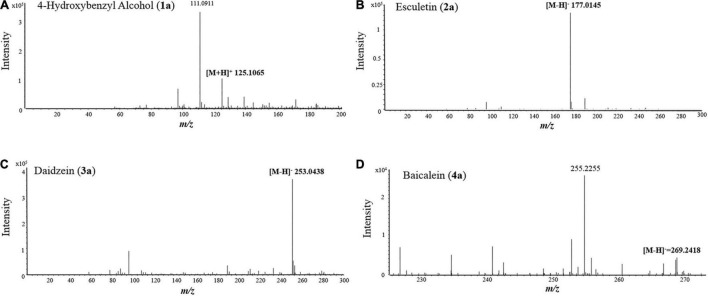
MS spectra of 4-hydroxybenzyl alcohol **(A)**, esculetin **(B)**, daidzein **(C)**, and baicalein **(D)**.

To test the ability and their potential applications of TtBgl3 in biotransformation of phenolic glycosides, we selected gastrodin, esculin and daidzin with β-glucoside bond and baicalin with β-glucuronide bond as the substrates of TtBgl3, and analyzed the conversion ability of recombinant TtBgl3 to phenolic glycosides (Figure 4). Based on HPLC analysis, the reaction system of phenolic glycosides produced new product peaks after TtBgl3 treatment (Figure 5). Each compound was analyzed by mass spectrometry (MS) to further confirm the structures of the transformation products (Figure 6). After comparing the molecular weight of each product, it was found that gastrodin, esculin, and daidzin all lost one glucose molecule and were converted into *p*-hydroxybenzyl alcohol, esculetin, and daidzein, respectively. In addition, baicalin lost a glucuronic acid molecule and converted to baicalein. By analyzing the chemical structure of four phenolic glycosides, we inferred that TtBgl3 acted on the β-glucoside/glucuronide bond of phenolic glycosides and transformed them into aglycon.

We optimized the enzyme concentration and transformation time at 37°C to further improve transformation efficiency (Supplementary Figures 3, 4). The optimal transformation parameters for three natural compounds were as follows: 60 U/mL for 8 h, 5 U/mL for 2 h, and 5 U/mL for 1 h at 37°C for gastrodin, esculin, and daidzin at the concentration of 500 g/mL with the transformation efficiency of 0.23, 0.92, and 0.31 mM/h, respectively. Under optimized conditions, the three substrates of gastrodin, esculin, and daidzin were completely hydrolyzed by TtBgl3, and the catalytic efficiency reached 100% (Table 3). The hydrolysis efficiency of baicalin was slightly lower, which was about 50%. When the substrate concentration was 500 g/mL, the yield of esculetin was the highest, 329.71 g/mL, while the yield of baicalein was 36.22 g/mL. Therefore, TtBgl3 not only acted on the β-glucoside bond of phenolic glycosides, but also acted on the β-glucuronide bond of phenolic glycosides, and the former was better.

**TABLE 3:** Transformation of different phenolic glycosides by recombinant TtBgl3.

Substrate	Concentration (μg/mL)	Transformation rate (%)	Products content (μg/mL)
Gastrodin	100	100	23.66 ± 0.15
	500	100	228.23 ± 8.76
Esculin	100	100	52.98 ± 0.39
	500	100	329.71 ± 2.26
Daidzin	100	100	29.63 ± 0.003
	500	100	78.92 ± 0.07
Baicalin	100	50.34 ± 1.94	0.26 ± 0.05
	500	49.35 ± 0.19	36.22 ± 0.25

### Homology Modeling and Substrate Docking

**FIGURE 7:**
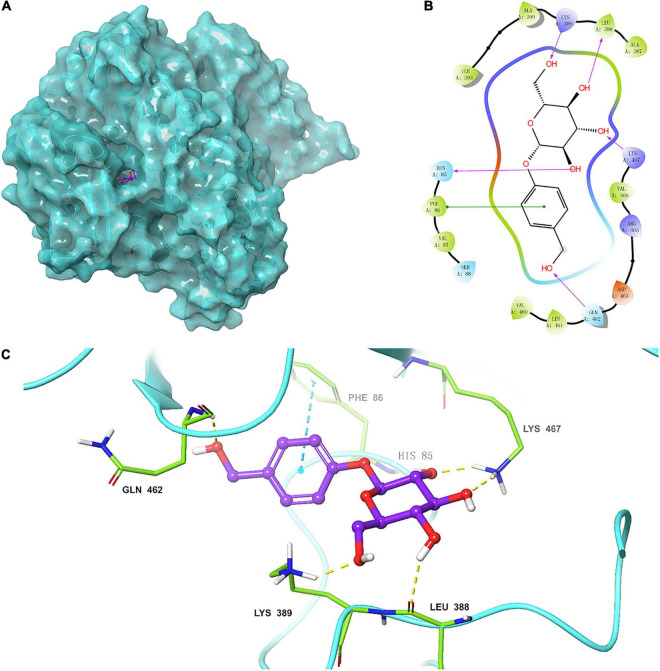
**(A)** Homologous model of TtBgl3 bound with gastrodin (1). The crystal structure of TtBgl3 (PDB accession number 3abz.1.A) was used as a template for homology modeling. **(B)** Residues of TtBgl3 interact with gastrodin (1). The hydrogen bond is shown as purple arrow. **(C)** Surface representation of the hydrophobic region of the TtBgl3 catalytic pocket. The hydrogen bond is shown by yellow dashed lines.

The 3D structure of TtBgl3 was established by online homologous modeling of the SWISS-MODEL database to predict the structure of TtBgl3. The QSQE, Seq identity, and GMQE were 0.4, 41.73, and 0.73, respectively, indicating that the structural model was reliable. As shown in the structural model (Figure 7A), the enzyme contained three domains (β/α)8-barrel domain, α/β sandwich domain, and fibronectin type III domain.

The modular docking procedure was used to analyze the binding sites of different phenolic glycosides with the recombinant TtBgl3 to understand the substrate specificity of the TtBgl3 (Figures 7B,C). The binding energies of proteins and small molecules were calculated using AutoDock Vina 1.1.2 software. After docking, the absolute values of binding constants of gastrodin, esculin, and daidzin was 7.9, 8.2, and 9.1, respectively. The substrates gastrodin, esculin, and daidzin could dock into the catalytic cleft of TtBgl3 well when the absolute values of binding constants were more than 7. These substrates were mainly combined with enzymes by hydrogen bonding, although the structures of the compounds were different. The substrate gastrodin containing a β-glucoside bond had two enzyme catalytic sites. It was speculated that the two residues of His85 and Lys467 were the enzyme-binding sites of the substrate containing a β-glucoside bond (Figures 7B,C). According to the β-glucosidase activity assays, the *H85A* mutant lost its catalytic activity with 8.83 U/mg protein, which is 2.62% of the activity of wild-type (337.4 U/mg protein) (Table 4). And the *K476L* mutant showed 23.83% of the activity of wild-type (Table 4). Further transformation study showed that the *H85A* and *K*476*L* mutants could cause a marked decrease in transformation rate with 0.18 and 6.41%, respectively, compared with complete transformation of gastrodin at the concentration of 500 μg/mL (Table 4). It was further confirmed that the two residues of His85 and Lys467 were essential for the catalytic hydrolysis activity of the recombinant TtBgl3.

**TABLE 4:** Enzyme activity and transformation of gastrodin catalyzed by wild-type (WT) TtBgl3 and the mutants of *H*85*A* and *K*476*L*.

	Enzyme activity (relative activity)*	Concentration (μg/mL)	Transformation rate (%)	4-Hydroxybenzyl alcohol (μg/mL)
WT	337.4 U/mg (100%)	100	100	23.66 ± 0.15
		500	100	228.23 ± 8.76
*H*85*A*	8.83 U/mg (2.62%)	100	0.11	0.12 ± 0.004
		500	0.18	0.95 ± 0.04
*K*476*L*	80.3 U/mg (23.8%)	100	4.72	4.85 ± 0.18
		500	6.41	32.60 ± 0.77

*The enzyme activity was determined using pNPG as the substrate.

## Discussion

*Trametes* genus and other species of white-rot fungi are important strains for wood degradation in nature and laccase production (Qin et al., 2018; Liu et al., 2019). A accumulating evidence indicates that white-rot fungi possess the whole GH enzyme system and can synthesize high amounts of enzymes for the degradation of lignocellulosic biomass including β-glucosidase (Qin et al., 2018; Liu et al., 2019). However, little work has been done to study the expression pattern, characteristics, and transformational properties of β-glucosidase and other GHs of white-rot fungi (Mallerman et al., 2015). The present study, found that 10 GH3 β-glucosidases was divided into three groups according to their expression pattern in the mixture of lignocellulose and glucose as carbon resources. Members of Group II were mainly expressed in the carbon source conversion stage, while members of Group I and III were the enzymes involved in lignocellulose degradation, indicating that these β-glucosidase isoenzymes might perform different physiological functions (Figure 1). Besides lignocellulose degradation, different members of β-glucosidase multigene family can play their unique roles in many biological processes, such as carbon recycling, cellulase gene induction, cell wall metabolism, host-pathogen interactions, and symbiotic association in microorganisms (Ahmed et al., 2017). Further purification and characterization of β-glucosidase isoenzymes of this strain might help reveal their detailed function.

Heterologous expression is one of the approaches to identify new β-glucosidases and other GH enzymes, and also increases their yield and overall productivity (Garvey et al., 2013). Until now, β-glucosidase genes from bacteria, yeast, and fungi have been cloned and expressed in *E. coli* and eukaryotic systems such as *S. cerevisiae*, *P. pastoris*, and *T*richoderma *reesei* (Ahmed et al., 2017). However, reports on the prokaryotical expression of β-glucosidase genes from white-rot fungus in *E. coli* are quite less due to several drawbacks such as formation of inclusion bodies, low secretion efficiency, and inability to perform post-translational modifications such as glycosylation (Ahmed et al., 2017), which is also confirmed by our study that only TtBgl3 coded by *T_trogii*_12914 was successfully expressed in a biochemically active form in *E. coli* (Figures 2, 3), while the other Group II protein coded by *T_trogii*_00539 was inactive. Until now, TtBgl3 is the first recombinant β-glucosidase of the *Trametes* genus, though the presence of β-glucosidase multigene family has been identified in many *Trametes* strains (Liu et al., 2019). Our results also added to the evidence that GH3 family members were the majority source of the recombinant fungal β-glucosidase (Ahmed et al., 2017).

β-Glucosidases differ in pH and temperature optima depending on their origin, and sources, and fungal β-glucosidases expressed in the eukaryotic expression system always have an acidic optimum pH and a higher optimum temperature above 60°C (Ahmed et al., 2017). The optimum pH and temperature for the recombinant TtBgl3, NfBGL595 of *Neosartorya fischeri* and Bgl3 of *Volvariella volvacea*, three fungal β-glucosidases expressed in *E. coli*, are pH 6.0 at 50°C, pH 6.0 at 40°C, and pH 6.4 at 50°C, respectively (Li et al., 2005; Ramachandran et al., 2012; Ahmed et al., 2017), indicating that the recombinant fungal β-glucosidases from *E. coli* preferred an intermediately neutral optimum pH and lower optimum temperature (Table 1). In addition, TtBgl3 was stable at a wide range of pH from 5.0 to 10.0 with more than 95% of maximum activity after 2 h treatment, which makes TtBgl3 a potential candidate in industrial bioconversion processes (Figure 3 and Table 1). *T. trogii* S0301, as a thermotolerant fungal strain, is considered a promising source of enzymes with improved stability (Yan et al., 2014). In order to adapt the higher temperature, other thermotolerant Bgls, besides TtBgl3, may exist in this strain.

Based on substrate specificity, β-glucosidases can be classified as cellobiase, aryl-β-D-glucosidase, and broad-substrate specificity β-glucosidase (Ahmed et al., 2017). In this study, TtBgl3 hydrolyzed only aryl-β-D-glucosides such as *p*NPG, and could be classified as aryl-β-glucosidases. Combined with its expression pattern during the carbon source conversion stage, it is possible that phenolic glycosides are the potential substrates of TtBgl3. To explore the potential substrates of TtBgl3, different type of substrates, including phenols (gastrodin), coumarins (esculin), isoflavones (daidzin), and flavonoids (baicalin), were chosen for transformation by TtBgl3.

After optimizing the hydrolysis conditions of three substrates containing β-glucoside bonds, the productivity for *p*-hydroxybenzyl alcohol, esculetin and daidzein was 0.23, 0.92, and 0.31 mM/h, respectively. It was worth noting that the yields of hydrolysis of gastrodin, esculin, and daidzin catalyzed by TtBgl3 reached 100%. Current reports showed that the hydrolysis rate of daidzin by T-Bgl from *Aspergillus terreus* and Bgl-CBM24 was about 95.78 and 85.22%, respectively (Yan et al., 2016; Chang et al., 2018), which was much lower than that of TtBgl3. In general, esculin is difficult to be hydrolyzed by β-glucosidase. For example, Os1Bglu4 from cytolinguistic rice only hydrolyzed a small amount of esculin (Rouyi et al., 2014). In this study, esculin was highly efficiently hydrolyzed by TtBgl3. Therefore, the recombinant TtBgl3 had greater advantages in the hydrolysis of isoflavone daidzin and esculin. Gastrodin was also completely hydrolyzed by TtBgl3, which was consistent with the rapid conversion of gastrodin into *p*-hydroxybenzyl alcohol by the intestinal microorganisms (Nepal et al., 2019). In addition, the recombinant TtBgl3 acted on not only the β-glucoside bond but also the β-glucuronide bond, and converted baicalin into baicalein. However, the hydrolysis rate of baicalin containing the β-glucuronide bond was relatively low, which reached 49.35% after 12 h. This was similar to the hydrolysis efficiency of Tpbgl1 from *Thermotoga petrophila* RKU-1 (Wu et al., 2018). In previous studies, three kinds of β-glucuronides, LbGus2, SbGus79, and SvGus79, were reported which specifically hydrolyzed the β-glucuronide bond (Sasaki et al., 2000; Huang et al., 2005; Sakurama et al., 2014).

Potential active sites (*His*85 and *Lys*467) in the acceptor-binding pocket were predicted and verified by mutation experiments to explore the catalytic mechanism of TtBgl3. In several reports, the kinetic and chemical modifications of β-glucosidase from *Ampullarium crossean* (Chen et al., 2000) and *T. reesei* (de la Mata et al., 1993) showed that His was directly involved in catalytic activity at the active site of the enzyme. In some GH3 glucosidases, histidine residue was generally considered as a potential proton donor. In addition, the addition of lysine to methionine (Met424Lys) increased the pH value of CelB-catalyzed glycosylation. Compared with the wild-type enzyme, both *Met*424*Lys* and *Phe*426*Tyr* mutants had better glycosylation activity at a lower lactose concentration (10–20%) (Hansson et al., 2001). It was suggested that His85 and Lys467 affected the catalytic activity of the enzyme.

## Conclusion

In summary, TtBgl3, a novel GH3 β-glucosidase of *T. trogii* S0301, was identified, prokaryotically expressed and biochemically characterized for the first time. The recombinant TtBgl3 preferred an intermediately neutral optimum pH and was stable at a wide range of pH. Moreover, TtBgl3 showed higher catalytic efficiency on β-glucoside bond of phenolic glycosides (gastrodin, esculin, and daidzin) than other known fungal counterparts with the 100% successful hydrolysis. Interestingly, the recombinant TtBgl3 is a dual-activity enzyme that has β-glucosidase and β-glucuronidase activity, due to the hydrolysis activity toward phenolic glycosides with a β-glucoside or β-glucuronide bond. These findings provide insights for the identification of novel GH3 β-glucosidases from *T. trogii* and other wood-rotting fungi. Furthermore, TtBgl3 might be applied as green and efficient biological catalysts in the deglycosylation of diverse phenolics to produce bioactive glycosides for drug discovery in the future.

## Data Availability Statement

The original contributions presented in the study are included in the article/Supplementary Material, further inquiries can be directed to the corresponding author.

## Author Contributions

YQ, YL, XY, and YZ: methodology, data curation, and writing—original draft. YH and EY: visualization and investigation. HX: methodology, software, and conceptualization. IC: software. JY: methodology, supervision, and writing—review and editing. All authors contributed to the article and approved the submitted version.

## Conflict of Interest

The authors declare that the research was conducted in the absence of any commercial or financial relationships that could be construed as a potential conflict of interest.

## Publisher’s Note

All claims expressed in this article are solely those of the authors and do not necessarily represent those of their affiliated organizations, or those of the publisher, the editors and the reviewers. Any product that may be evaluated in this article, or claim that may be made by its manufacturer, is not guaranteed or endorsed by the publisher.

## Footnotes

1
http://gsds.cbi.pku.edu.cn/

2
https://smart.embl.de

3
http://www.megasoftware.net/

4
https://www.swissmodel.expasy.org/

5
https://pubchem.ncbi.nlm.nih.gov/
