# Supplementary material for: Highly Efficient Biotransformation of Phenolic Glycosides Using a Recombinant β-Glucosidase From White Rot Fungus Trametes trogii
Source: Front Microbiol. 2022 May 18;13:762502. doi: 10.3389/fmicb.2022.762502 (PMC9158485; doi:10.3389/fmicb.2022.762502)
Supplement: Supplementary file 1 [file Data_Sheet_1.docx]

Supplementary Material


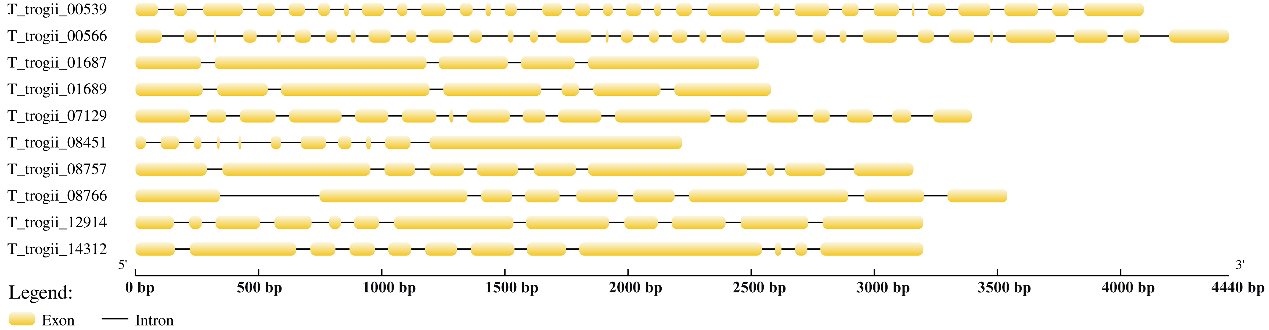


**Supplementary Figure 1.** Structural analysis of 10 GH3 genes in *Trametes trogii* S0301.


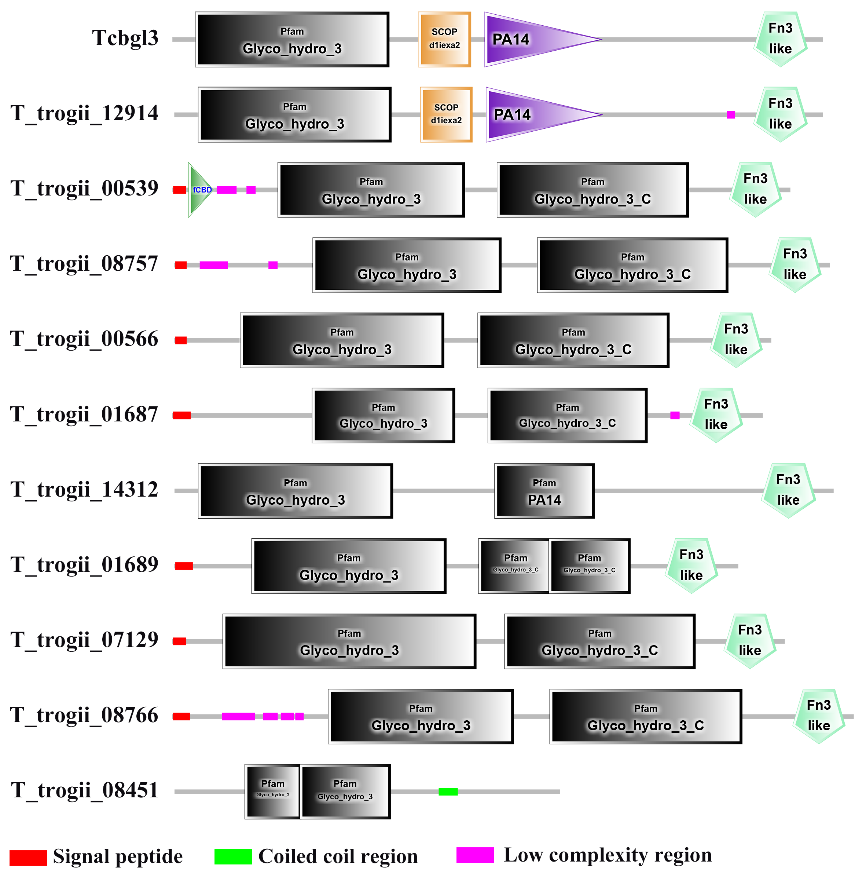


**Supplementary Figure 2.** Domain prediction and comparison analysis of 10 GH3 genes in *Trametes trogii* S0301.


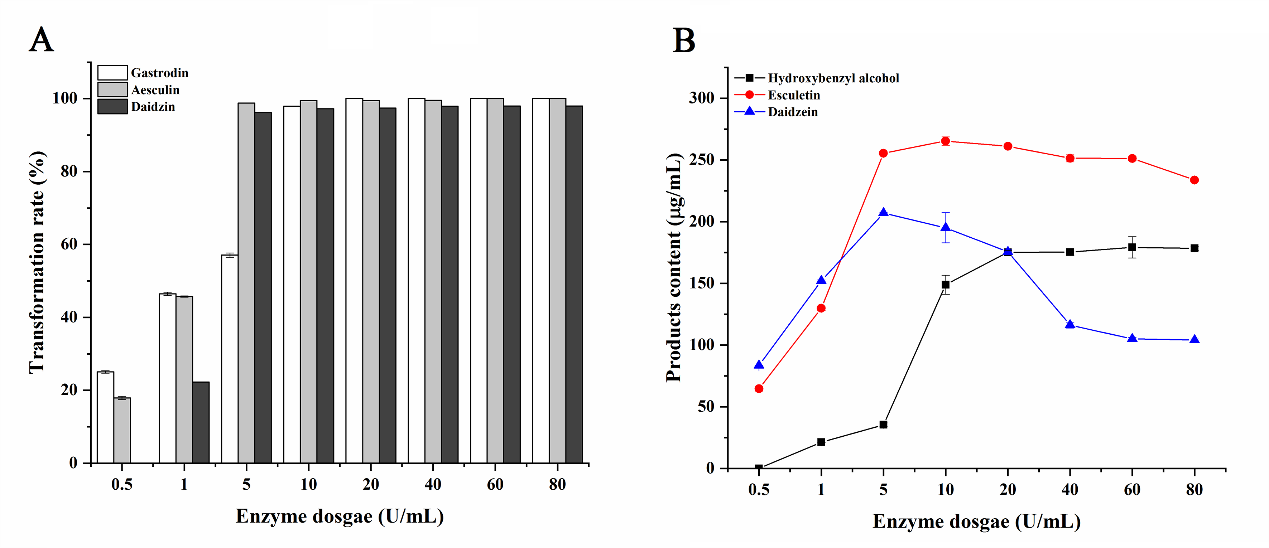


**Supplementary Figure 3** Analysis of transformation rate (A) and products contents (B) of phenolic glycosides with different concentration of recombinant TtBGL3 at 37°C for 12 h.


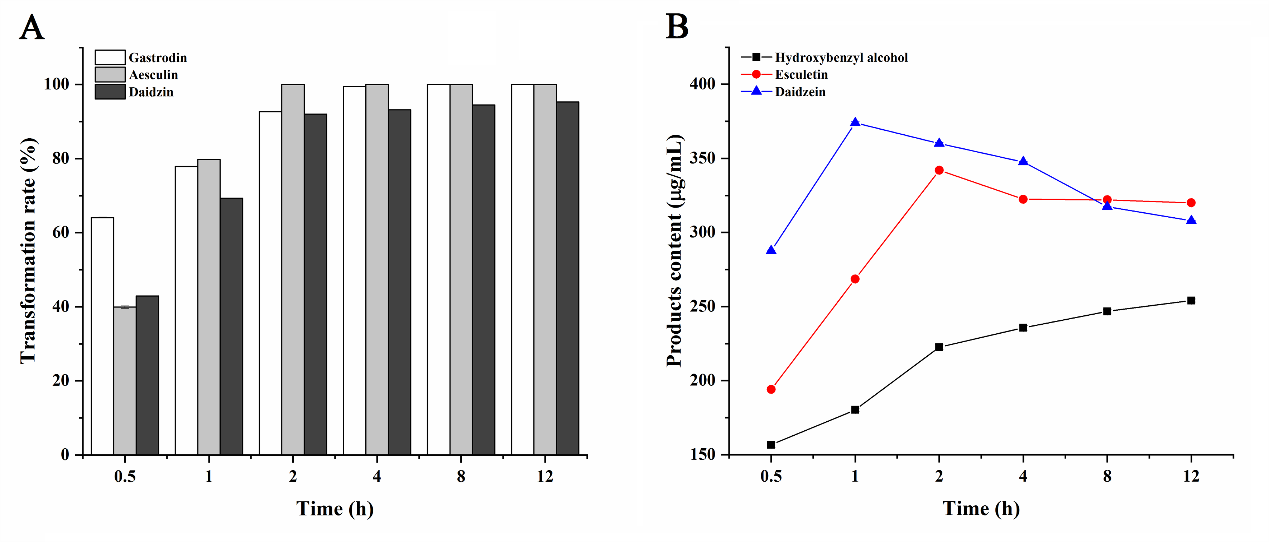


**Supplementary Figure 4** Analysis of transformation rate (A) and products contents (B) of phenolic glycosides with different incubation time after adding 10 U/mL recombinant TtBGL3 at 37°C.

**Table S1** The distribution of the putative signal peptides in of the GH3 gene family.

| **Transcript ID/**  **Genebank number** | **Amino Acid Sequence** | **Cleavage site** | **D-score** |
| --- | --- | --- | --- |
| **T_trogii_00539** | **MVFRLTASLLLLAGTVLG↓QS** | **18 and 19** | **0.6650** |
| **T_trogii_00566** | **MAVLALAPALVALVYALALGSA↓QS** | **22 and 23** | **0.4296** |
| **T_trogii_01687** | **MANGLRRLAALLAALVLGAASVQA↓YG** | **24 and 25** | **0.8426** |
| **T_trogii_01689** | **MANGLRRLAALLPALAVGAASVQA↓QA** | **24 and 25** | **0.4951** |
| **T_trogii_07129** | **MRALSFALLFLFSVGGYA↓SS** | **18 and 19** | **0.6354** |
| **T_trogii_08757** | **MRLSVFCALFAILANA↓KR** | **16 and 17** | **0.9176** |
| **T_trogii_08766** | **MALVLNTRLSALCALLLAVSASA↓QP** | **23 and 24** | **0.6960** |
| **T_trogii_14312** | **-** | **-** | **0.0032** |
| **T_trogii_12914** | **-** | **-** | **0.0012** |
| **T_trogii_08451** | **-** | **-** | **0.0012** |

-
